# On the cytotoxicity of HCR-NTPase in the neuroblastoma cell line SH-SY5Y

**DOI:** 10.1186/1756-0500-2-102

**Published:** 2009-06-11

**Authors:** Markus Pasdziernik, Barbara Kaltschmidt, Christian Kaltschmidt, Claudia Klinger, Michael Kaufmann

**Affiliations:** 1 The Protein Chemistry Group, Witten/Herdecke University, Stockumer Str 10, 58453 Witten, Germany; 2 Institute for Neurobiochemistry, Witten/Herdecke University, Stockumer Str 10, 58453 Witten, Germany; 3 Dept. of Cell Biology, Bielefeld University, Universitätsstr 25, 33501 Bielefeld, Germany

## Abstract

**Background:**

The human cancer-related nucleoside triphosphatase (HCR-NTPase) is overexpressed in several tumour tissues including neuroblastoma. HCR-NTPase is an enzyme exhibiting a slow *in vitro *activity in hydrolysing nucleosidetriphosphates. However, its *in vivo *function is still unknown. To learn more about the physiological role of HCR-NTPase, we both overexpressed and silenced it in the neuroblastoma cell line SH-SY5Y.

**Findings:**

No effect was observed when the expression of endogenously expressed HCR-NTPase in the cells was silenced by RNA interference. On the other hand, overexpression of HCR-NTPase led to cytotoxicity of the protein in SH-SY5Y cells. Even if the catalytic essential amino acid glutamate 114 was replaced by alanine (E114A-HCR-NTPase), the protein remained cytotoxic. The results could be confirmed by successfully rescuing the cells via RNA interference.

**Conclusion:**

Although expressed in several tumours, at least in SH-SY5Y, HCR-NTPase is not essential for the cells to survive. Increased levels of the protein lead to cytotoxicity due to physical intracellular interactions rather than hydrolysis of nucleosidetriphosphates by its intrinsic residual enzymatic activity.

## Reseach hypothesis

A screen of the cancer genome anatomy project (CGAP) database [1,2] for expressed sequence tags that compared to normal tissue are highly expressed in human tumours revealed the human cancer-related nucleoside triphosphatase (HCR-NTPase) [3]. HCR-NTPase, the gene product of the mRNA NM_032324 (synonyms: MGC13186, LOC84284, C1orf57, GI:14150100) is described to exhibit an increased expression profile in liver cholangiocarcinoma when compared to normal tissue [3]. In addition, as retrieved from SOURCE [4,5] when this project was initiated, HCR-NTPase was stated to be expressed in many human tumours, several of them located in the brain such as medullablastoma, glioblastoma, and neuroblastoma.

A homology search using BLAST [6,7] retrieved significant sequence homologies between HCR-NTPase and proteins assigned to COG1618 of the COG database [8]. As a representative of COG1618 proteins, aaTHEP1 from the hyperthermophilic bacterium *Aquifex aeolicus *was biochemically characterised [9] and its crystal structure was resolved [10]. Both HCR-NTPase and aaTHEP1 hydrolyse ATP and GTP *in vitro *with K_m _in the micromolar range and k_cat _in the range between 5 and 9 × 10^-3 ^s^-1 ^corresponding to approximately 20–30 molecules hydrolysed per hour and enzyme molecule. In addition, the proteins resemble each other in terms of structure. They belong to the class of P-loop NTPases, and their regular secondary structures can be superimposed with a backbone RMSD of 2.8 Å [3]. Thus, aaTHEP1 and consequently COG1618 proteins may serve as model systems for HCR-NTPase.

Analysing the phylogenetic distribution of COG1618 proteins by phylogenetic COG ranking [11,12] reveals that they are absent from almost all mesophiles and present in all thermophiles, most of them archeae. In addition, HCR-ATPase is present in many eucarya analysed thus far. Therefore, HCR-ATPase and COG1618 proteins are at least very similar to PACE proteins *i. e*. proteins from archaea without assigned function that are conserved in eukarya as described by Matte-Tailliez *et al*. [13]. The authors argue that most of the PACE proteins are informational proteins and could be of strong biomedical interest.

The aim of this study was to obtain further insight into the role of HCR-NTPase in tumour cells. For that purpose we investigated the phenotype of the neuroblastoma cell line SH-SY5Y [14] as a model system for brain tumours under both conditions overexpressing and silencing HCR-NTPase.

## Methods

### Eucaryotic cell culture and transfections

SH-SY5Y neuroblastoma cells [14] were obtained from ATCC, Manassas, USA and grown at 37°C and 5% CO_2 _in Dulbecco's modified Eagle's Medium containing 1% penicillin/streptomycin and 15% fetal calf serum (growth medium; GIBCO, Eggenstein, Germany) in a humidified incubator. To keep the cells in logarithmic growth, confluent cells were washed with phosphate buffered saline (PBS), detached by 0.05% Trypsin and 0.5 mM EDTA in PBS, followed by washing and diluting them in fresh growth medium. 10^6 ^cells were transfected with 2 μg Plasmid by electroporation in 50 μl of Cell Line Nucleofector V solution (Amaxa, Cologne, Germany). After transfection, cells were washed in growth medium, transferred to 6-well plates and judged microscopically after 24 h of incubation at 37°C and 5% CO_2 _using an Axiovert100 microscope (Zeiss, Oberkochen, Germany). Red fluorescent cells were analysed at 584 nm and green fluorescent cells at 482 nm excitation. Three different fields of view were evaluated for each experiment and all experiments were performed independently in triplicate.

### Silencing HCR-NTPase in SH-SY5Y by RNA interference

The expression of HCR-NTPase in SH-SY5Y cells was blocked via RNA interference [15] using pSilencer1.0-U6 (Ambion, Darmstadt, Germany). shRNA coding inserts flanked by ApaI and EcoRI restriction sites were constructed by phosphorylating 25 pmol of oligonucleotides (Operon, Cologne, Germany) using T4 poynucleotide kinase, followed by assembling complementary pairs by incubation for 5 min at 100°C and 60 min at 37°C. pSilencer1.0-U6 sequentially was cut by ApaI (Fermentas, St. Leon-Rot, Germany) and EcoRI (Fermentas, St. Leon-Rot, Germany), the inserts were inserted and the plasmids were amplified in *E. coli *SURE (Stratagene, San Diego, USA). Expression of HCR-NTPase was blocked by transfecting SH-SY5Y cells with purified plasmids. The following pairs of oligonucleotides were used:

siRNA1:

5'-ATC CAT AAA GCC AGT GAT TCT CAA GAG AAA TCA CTG GCT TTA TGG ATC ATT TTT T-3' and

5'-AAT TAA AAA ATG ATC CAT AAA GCC AGT GAT TTC TCT TGA GAA TCA CTG GCT TTA TGG ATG GCC-3'

siRNA2:

5'-AGA GCC TCC ACC TGG AAT TCT CAA GAG AAA TTC CAG GTG GAG GCT CTA ATT TTT T-3' and

5'-AAT TAA AAA ATT AGA GCC TCC ACC TGG AAT TTC TCT TGA GAA TTC CAG GTG GAG GCT CTG GCC-3'

siRNA3:

5'-GAA TGC CGA CTG CAG CAT TCT CAA GAG AAA TGC TGC AGT CGG CAT TCC TTT TTT T-3' and

5'-AAT TAA AAA AAG GAA TGC CGA CTG CAG CAT TTC TCT TGA GAA TGC TGC AGT CGG CAT TCG GCC-3'

siRNA4:

5'-TTC CTA AAG GAA AGC CAT TCT CAA GAG AAA TGG CTT TCC TTT AGG AAC TTT TTT T-3' and

5'-AAT TAA AAA AAG TTC CTA AAG GAA AGC CAT TTC TCT TGA GAA TGG CTT TCC TTT AGG AAG GCC-3'

### Overexpressing HCR-NTPase in SH-SY5Y cells

HCR-NTPase cDNA was obtained from RZPD, Berlin, Germany. The gene was amplified and cloned into pET101/D-TOPO yielding pET101/D-TOPO/HCR-NTPase to express the protein in *E. coli*. This construct also served as the basis for all further cloning steps. HCR-NTPase was overexpressed using the plasmid pRc/CMV (Invitrogen, Karlsruhe, Germany). The gene was amplified from pET101/D-TOPO/HCR-NTPase using the primers

5'-AAA AGC TTA TGG CCC GGC ACG TGT TCC-3' and

5'-AAA ATC TAG ATC ACT TCC TGC TGC TCT G-3'.

The PCR-fragment was digested sequentially by XbaI (Fermentas, St. Leon-Rot, Germany) and HindIII (Fermentas, St. Leon-Rot, Germany), inserted into pRc/CMV and amplified in *E. coli *TOP10 (Invitrogen, Karlsruhe, Germany).

HCR-NTPase as a fusion with red fluorescent protein [16] (HCR-NTPase-RFP) was overexpressed using the plasmid pmaxFP-Red-C (Amaxa, Cologne, Germany). The gene was amplified from pET101/D-TOPO/HCR-NTPase using the primers

5'-AAA AGA TCT GGA GGA GGA GGA ATG GCC CGG CAC GTG TTC-3' and

5'-AAG GTA CCT CAC TTC CTG CTG CTC TG-3'.

Before digesting with BglII (Fermentas, St. Leon-Rot, Germany) and KpnI (Fermentas, St. Leon-Rot, Germany), the PCR-fragment was inserted into pCR2.1-TOPO (Invitrogen, Karlsruhe, Germany). The resulting fragment was inserted into pmaxFP-Red-C and amplified in *E. coli *TOP10. As a control for the efficiency of transfection, pmaxGFP (QIAGEN) was used. HCR-NTPase was overexpressed by transfecting SH-SY5Y cells with purified plasmids.

### Construction of a E114A-HCR-NTPase mutant in pRc/CMV

An enzymatically inactive HCR-NTPase mutant was constructed by mutating a conserved catalytic glutamate [17]. For that purpose, E114 of HCR-NTPase in pRc/CMV was replaced by an alanin using the Phusion™ Site-Directed Mutagenesis Kit (Finnzymes, Espoo, Finland) according to the instructions of the manufacturer. The utilised phosphorylated primers were

5'-P-CGT CAT CGA TGC GAT TGG GAA GA-3' and

5'-P-CAC ACT CTT TGC CCT GGG CCA-3'.

### Inhibition of apoptosis

Apoptosis was inhibited by adding the caspase inhibitor Z-VAD-FMK (Promega, Mannheim) to the culture medium directly after transfection at a final concentration of 20 μM.

### Bioinformatics

ClustalW-alignments were performed as previously described [18,19] and for the graphical representation, Genedoc was used [20].

All investigations have been performed in accordance to German regulatory affairs.

## Results

### Loss-of-function: silencing HCR-NTPase by RNA interference

**Figure 1:**
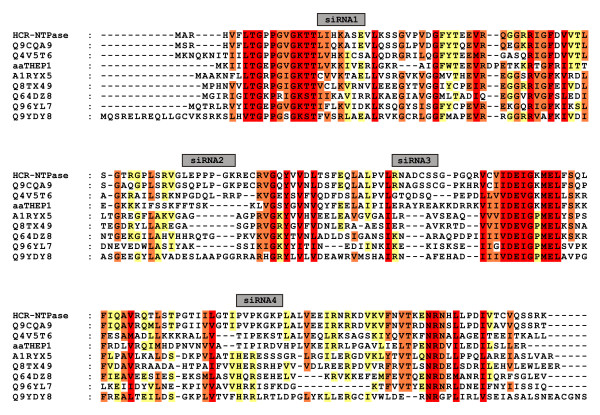
**Positions of siRNAs used to silence HCR-NTPase in SH-SY5Y**. ClustalW-alignment of the eight most diverse sequences out of a group of 29 species that have been found to contain Walker A and B motifs similar to those of HCR-NTPase [3]. The sequences were from *Homo sapiens*, *Mus musculus*, *Drosophila melanogaster*, *Aquifex aeolicus *(aaTHEP1), *Thermophilum pendens*, *Methanopyrus kandleri*, an uncultured archaeon, *Sulfolobus tokodaii*, and *Aeropyrum pernix*, respectively.

**Figure 2:**
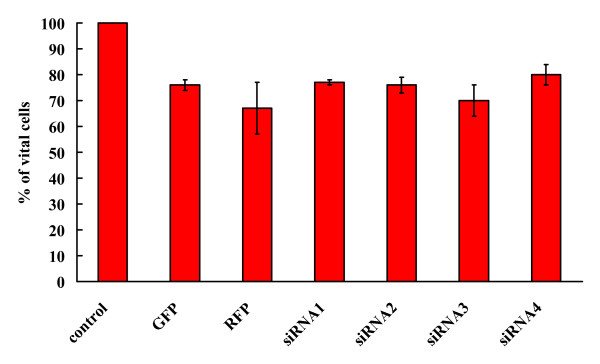
**Silencing HCR-NTPase in SH-SY5Y has no effect**. Normalised cell counts (in % of untransfected control) of SH-SY5Y transfected with plasmids containing GFP, RFP, siRNA1, siRNA2, siRNA3, and siRNA4, respectively. The data for the GFP- and RFP-controls are the same as those shown in table 1. The error bars indicate the standard deviations of at least 3 independently performed experiments.

Since HCR-NTPase is overexpressed in neuroblastoma we were interested in the phenotype of SH-SY5Y if the intrinsic expression of HCR-NTPase was suppressed. For that purpose we constructed plasmids containing siRNAs directed to the regions within HCR-NTPase that are shown in figure 1. Untreated cells and those transfected with plasmids containing siRNAs were analysed by their cell counts as judged via phase contrast microscopy. To show the effect caused by the transfection procedure, cells transfected with constructs encoding GFP and RFP were included as controls. As can be seen in figure 2, the cell counts are reduced by about one fourth by the transfection procedure alone. However, compared to the GFP- and RFP-controls the cells remain mainly unaffected regardless of which siRNA constructs were introduced.

### Gain-of-function: overexpressing HCR-NTPase in SH-SY5Y

**Figure 3:**
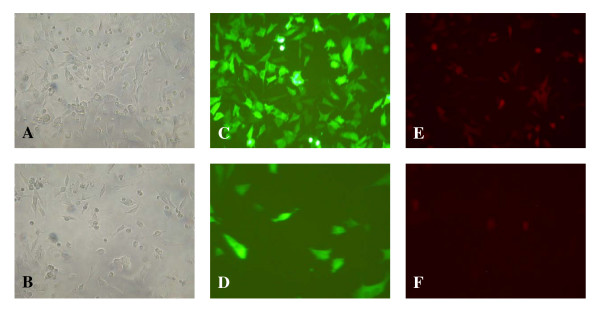
**Cytotoxicity of HCR-NTPase expressed in SH-SY5Y**. Phase contrast and fluorescence images of SH-SY5Y cells are shown. The cells were either untreated (A) or transfected with Plasmids encoding HCR-NTPase (B), GFP (C), GFP + HCR-NTPase (D), RFP (E), and an HCR-NTPase-RFP fusion product (F).

To judge the effect of overexpressed HCR-NTPase in SH-SY5Y cells, transfections with different expression plasmids were performed. The cultures were then analysed by both phase contrast and fluorescence microscopy. Compared to the controls, HCR-NTPase, a combination of HCR-NTPase and GFP as well as a fusion product between HCR-NTPase and RFP (HCR-NTPase-RFP) gave rise to significant fewer cell counts demonstrating HCR-NTPase mediated cytotoxicity (Figure 3). Table 1 quantitatively shows the cytotoxic action of HCR-NTPase. The efficiency of transfection was judged by quantifying fluorescent cells after being transfected with GFP and RFP containing plasmids. Compared to the total number of cells visible by phase contrast microscopy, those also showing fluorescence were 80% and 64% for GFP and RFP, respectively. As can be seen, far less than half of the cells survived if transfected with either HCR-NTPase or HCR-NTPase-RFP. Moreover, the fraction of red fluorescent cells after transfection with HCR-NTPase-RFP was negligible, indicating that the vast majority of surviving cells were untransfected rather than unaffected by a successful transfection. Similar results were obtained when the cells were cotransfected with HCR-NTPase and GFP supporting the presumption that the surviving cells contained no plasmids.

**Table 1:** Quantitative analysis of the cytotoxicity of HCR-NTPase expressed in SH-SY5Y

	Total	red	green
Control	100	-	-
GFP^6^	76 (2)	-	61 (2)
RFP^6^	67 (10)	43 (8)	-
HCR-NTPase^9^	31 (5)	-	-
HCR-NTPase-RFP^3^	24 (3)	1 (1)	-
GFP + HCR-NTPase^6^	30 (9)	-	7 (2)
GFP + HCR-NTPase-RFP^3^	30 (4)	1 (1)	6 (2)

SH-SY5Y cells were transfected with plasmids containing green fluorescent protein (GFP), red fluorescent protein (RFP), HCR-NTPase, or a fusion of HCR-NTPase with red fluorescent protein (HCR-NTPase-RFP) as inserts. The percentage of total cells normalised to 100 for untreated cells (control) as microscopically judged by phase contrast (total), red fluorescence (red), and green fluorescence (green) are given. In parenthesis the standard deviations, and as uppercase numbers the number of independently performed experiments are stated.

### Rescue of SH-SY5Y cells by RNA interference

**Figure 4:**
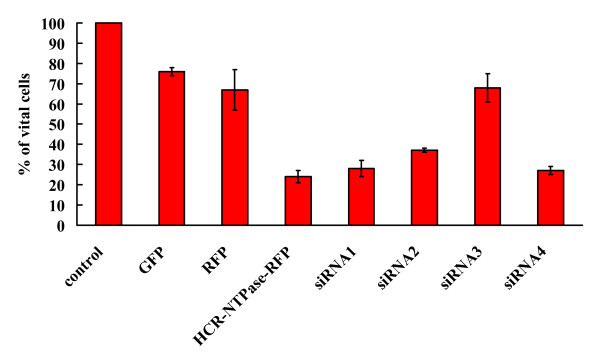
**Rescue of SH-SY5Y cells by RNA-interference**. Normalised cell counts (in % of untransfected control) of SH-SY5Y transfected with plasmids encoding a fusion protein between HCR-NTPase and RFP alone (HCR-NTPase-RFP) or cotransfected with siRNA1, siRNA2, siRNA3, and siRNA4, respectively. Compared to the control, less than 1% of cells containing the HCR-NTPase-RFP fusion protein could be detected by fluorescence microscopy (data not shown). The data for the GFP-, RFP- and HCR-NTPase-RFP-controls are the same as those shown in table 1. The error bars indicate the standard deviations of at least 3 independently performed experiments.

To confirm that the expression of HCR-NTPase was indeed the cause of the observed cell death after transfection with HCR-NTPase containing plasmids, we tried to rescue the cells by means of RNA interference. Whereas siRNA1 and siRNA4 showed no effect, siRNA2 significantly increased the survival rate and siRNA3 was even able to completely rescue the cells yielding the same cell counts observed when the cells were transfected with plasmids encoding solely RFP or GFP (figure 4).

### The E114A-HCR-NTPase mutant

**Figure 5:**
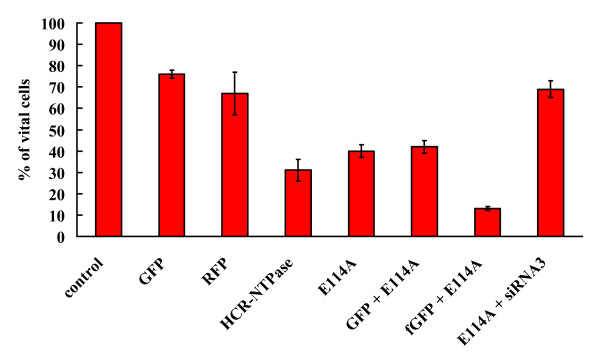
**Cytotoxicity of the E114A-HCR-NTPase mutant and rescue of SH-SY5Y cells by siRNA3**. Normalised cell counts (in % of untransfected control) of SH-SY5Y transfected with plasmids encoding the E114A-HCR-NTPase mutant (E114A) alone or cotransfected with either GFP or siRNA3. Whereas fGFP + E114A were analysed by fluorescence microscopy, all other cells were counted via phase contrast microscopy. The data for the GFP-, RFP- and HCR-NTPase-controls are the same as those shown in table 1. The error bars indicate the standard deviations of at least 3 independently performed experiments.

HCR-NTPase belongs to the ASCE (additional strand, catalytic E) class of NTPases [3]. In ASCE NTPases, NTP-hydrolysis typically depends on a conserved catalytic glutamate (E114 in HCR-NTPase and E107 in aaTHEP1). This essential glutamate is described to prime a water molecule for a nucleophilic attack on the γ-phosphate [17,21]. Consequently, a mutant protein lacking this essential glutamate is expected to be catalytically inactive. Such a mutant was then suitable to decide whether unspecific intracellular hydrolysis of nucleotides or the physical interaction with other intracellular compounds is responsible for the observed cytotoxicity. Hence, we constructed the E114A-HCR-NTPase mutant and expressed it in SH-SY5Y cells. As shown in figure 5, the effect of E114A-HCR-NTPase on SH-SY5Y cells resembles that one observed with the wild type protein. Although to a slightly lesser degree, the cells are killed by E114A-HCR-NTPase and can be rescued by siRNA3 mediated RNA interference excluding unspecific NTP-hydrolysis as the cause of cytotoxicity. The cells died due to apoptosis as could be seen morphologically. In addition, as judged by green fluorescence, we successfully rescued them by the caspase inhibitor Z-VAD-FMK (table 2).

**Table 2:** Effect of Z-VAD-FMK on the cytotoxicity induced by HCR-NTPase and E114A-HCR-NTPase in SH-SY5Y

Treatment	Fluorescent cells
GFP (control)	100 (20)
GFP + Z-VAD-FMK	93 (15)
GFP + HCR-NTPase	4 (4)
GFP + HCR-NTPase + Z-VAD-FMK	91 (21)
GFP + E114A-HCR-NTPase	79 (12)
GFP + E114A-HCR-NTPase + Z-VAD-FMK	95 (17)

SH-SY5Y cells were transfected with plasmids containing green fluorescent protein (GFP), HCR-NTPase or E114A-HCR-NTPase. The percentage of total cells normalised to 100 for cells only treated with GFP (control) as microscopically judged by green fluorescence are given with and without adding the caspase inhibitor Z-VAD-FMK. In parenthesis the standard deviations are stated. Three experiments were performed independently.

## Competing interests

All authors disclose any financial and personal relationships with other people or organisations that could inappropriately influence (bias) their work.

## Authors' contributions

MP carried out the cell culture studies and participated in the cloning experiments. BK designed the siRNA constructs and participated in the design of the study. CKA and MK conceived of the study and participated in its design. CKL carried out the molecular biology experiments and participated in the cloning steps. MK drafted the manuscript. All authors read and approved the final manuscript.
